# BioTIME: A database of biodiversity time series for the Anthropocene

**DOI:** 10.1111/geb.12729

**Published:** 2018-07-24

**Authors:** Maria Dornelas, Laura H. Antão, Faye Moyes, Amanda E. Bates, Anne E. Magurran, Dušan Adam, Asem A. Akhmetzhanova, Ward Appeltans, José Manuel Arcos, Haley Arnold, Narayanan Ayyappan, Gal Badihi, Andrew H. Baird, Miguel Barbosa, Tiago Egydio Barreto, Claus Bässler, Alecia Bellgrove, Jonathan Belmaker, Lisandro Benedetti‐Cecchi, Brian J. Bett, Anne D. Bjorkman, Magdalena Błażewicz, Shane A. Blowes, Christopher P. Bloch, Timothy C. Bonebrake, Susan Boyd, Matt Bradford, Andrew J. Brooks, James H. Brown, Helge Bruelheide, Phaedra Budy, Fernando Carvalho, Edward Castañeda‐Moya, Chaolun Allen Chen, John F. Chamblee, Tory J. Chase, Laura Siegwart Collier, Sharon K. Collinge, Richard Condit, Elisabeth J. Cooper, J. Hans C. Cornelissen, Unai Cotano, Shannan Kyle Crow, Gabriella Damasceno, Claire H. Davies, Robert A. Davis, Frank P. Day, Steven Degraer, Tim S. Doherty, Timothy E. Dunn, Giselda Durigan, J. Emmett Duffy, Dor Edelist, Graham J. Edgar, Robin Elahi, Sarah C. Elmendorf, Anders Enemar, S. K. Morgan Ernest, Rubén Escribano, Marc Estiarte, Brian S. Evans, Tung‐Yung Fan, Fabiano Turini Farah, Luiz Loureiro Fernandes, Fábio Z. Farneda, Alessandra Fidelis, Robert Fitt, Anna Maria Fosaa, Geraldo Antonio Daher Correa Franco, Grace E. Frank, William R. Fraser, Hernando García, Roberto Cazzolla Gatti, Or Givan, Elizabeth Gorgone‐Barbosa, William A. Gould, Corinna Gries, Gary D. Grossman, Julio R. Gutierréz, Stephen Hale, Mark E. Harmon, John Harte, Gary Haskins, Donald L. Henshaw, Luise Hermanutz, Pamela Hidalgo, Pedro Higuchi, Andrew Hoey, Gert Van Hoey, Annika Hofgaard, Kristen Holeck, Robert D. Hollister, Richard Holmes, Mia Hoogenboom, Chih‐hao Hsieh, Stephen P. Hubbell, Falk Huettmann, Christine L. Huffard, Allen H. Hurlbert, Natália Macedo Ivanauskas, David Janík, Ute Jandt, Anna Jażdżewska, Tore Johannessen, Jill Johnstone, Julia Jones, Faith A. M. Jones, Jungwon Kang, Tasrif Kartawijaya, Erin C. Keeley, Douglas A. Kelt, Rebecca Kinnear, Kari Klanderud, Halvor Knutsen, Christopher C. Koenig, Alessandra R. Kortz, Kamil Král, Linda A. Kuhnz, Chao‐Yang Kuo, David J. Kushner, Claire Laguionie‐Marchais, Lesley T. Lancaster, Cheol Min Lee, Jonathan S. Lefcheck, Esther Lévesque, David Lightfoot, Francisco Lloret, John D. Lloyd, Adrià López‐Baucells, Maite Louzao, Joshua S. Madin, Borgþór Magnússon, Shahar Malamud, Iain Matthews, Kent P. McFarland, Brian McGill, Diane McKnight, William O. McLarney, Jason Meador, Peter L. Meserve, Daniel J. Metcalfe, Christoph F. J. Meyer, Anders Michelsen, Nataliya Milchakova, Tom Moens, Even Moland, Jon Moore, Carolina Mathias Moreira, Jörg Müller, Grace Murphy, Isla H. Myers‐Smith, Randall W. Myster, Andrew Naumov, Francis Neat, James A. Nelson, Michael Paul Nelson, Stephen F. Newton, Natalia Norden, Jeffrey C. Oliver, Esben M. Olsen, Vladimir G. Onipchenko, Krzysztof Pabis, Robert J. Pabst, Alain Paquette, Sinta Pardede, David M. Paterson, Raphaël Pélissier, Josep Peñuelas, Alejandro Pérez‐Matus, Oscar Pizarro, Francesco Pomati, Eric Post, Herbert H. T. Prins, John C. Priscu, Pieter Provoost, Kathleen L. Prudic, Erkki Pulliainen, B. R. Ramesh, Olivia Mendivil Ramos, Andrew Rassweiler, Jose Eduardo Rebelo, Daniel C. Reed, Peter B. Reich, Suzanne M. Remillard, Anthony J. Richardson, J. Paul Richardson, Itai van Rijn, Ricardo Rocha, Victor H. Rivera‐Monroy, Christian Rixen, Kevin P. Robinson, Ricardo Ribeiro Rodrigues, Denise de Cerqueira Rossa‐Feres, Lars Rudstam, Henry Ruhl, Catalina S. Ruz, Erica M. Sampaio, Nancy Rybicki, Andrew Rypel, Sofia Sal, Beatriz Salgado, Flavio A. M. Santos, Ana Paula Savassi‐Coutinho, Sara Scanga, Jochen Schmidt, Robert Schooley, Fakhrizal Setiawan, Kwang‐Tsao Shao, Gaius R. Shaver, Sally Sherman, Thomas W. Sherry, Jacek Siciński, Caya Sievers, Ana Carolina da Silva, Fernando Rodrigues da Silva, Fabio L. Silveira, Jasper Slingsby, Tracey Smart, Sara J. Snell, Nadejda A. Soudzilovskaia, Gabriel B. G. Souza, Flaviana Maluf Souza, Vinícius Castro Souza, Christopher D. Stallings, Rowan Stanforth, Emily H. Stanley, José Mauro Sterza, Maarten Stevens, Rick Stuart‐Smith, Yzel Rondon Suarez, Sarah Supp, Jorge Yoshio Tamashiro, Sukmaraharja Tarigan, Gary P. Thiede, Simon Thorn, Anne Tolvanen, Maria Teresa Zugliani Toniato, Ørjan Totland, Robert R. Twilley, Gediminas Vaitkus, Nelson Valdivia, Martha Isabel Vallejo, Thomas J. Valone, Carl Van Colen, Jan Vanaverbeke, Fabio Venturoli, Hans M. Verheye, Marcelo Vianna, Rui P. Vieira, Tomáš Vrška, Con Quang Vu, Lien Van Vu, Robert B. Waide, Conor Waldock, Dave Watts, Sara Webb, Tomasz Wesołowski, Ethan P. White, Claire E. Widdicombe, Dustin Wilgers, Richard Williams, Stefan B. Williams, Mark Williamson, Michael R. Willig, Trevor J. Willis, Sonja Wipf, Kerry D. Woods, Eric J. Woehler, Kyle Zawada, Michael L. Zettler, Thomas Hickler

**Affiliations:** ^1^ Centre for Biological Diversity and Scottish Oceans Institute, School of Biology, University of St. Andrews St Andrews United Kingdom; ^2^ Department of Biology and CESAM Universidade de Aveiro, Campus Universitário de Santiago Aveiro Portugal; ^3^ National Oceanography Centre, University of Southampton Waterfront Campus Southampton United Kingdom; ^4^ Department of Ocean Sciences, Memorial University of Newfoundland St John's Newfoundland and Labrador Canada; ^5^ Department of Forest Ecology, Silva Tarouca Research Institute Brno Czech Republic; ^6^ Department of Geobotany, Faculty of Biology Moscow State University Moscow Russia; ^7^ UNESCO, Intergovernmental Oceanographic Commission, IOC Project Office for IODE Oostende Belgium; ^8^ SEO/BirdLife, Marine Programme Barcelona Spain; ^9^ Department of Ecology French Institute of Pondicherry Puducherry India; ^10^ ARC Centre of Excellence for Coral Reef Studies, James Cook University Townsville Queensland Australia; ^11^ Laboratório de Ecologia e Restauração Florestal, Fundação Espaço Eco, Piracicaba, São Paulo Brazil; ^12^ Bavarian Forest National Park Grafenau Germany; ^13^ School of Life and Environmental Sciences Centre for Integrative Ecology, Deakin University Warrnambool Victoria Australia; ^14^ School of Zoology, George S. Wise Faculty of Life Sciences Tel Aviv University Tel Aviv Israel; ^15^ Department of Biology University of Pisa Pisa, CoNISMa Italy; ^16^ Section for Ecoinformatics and Biodiversity, Department of Bioscience Aarhus University Aarhus Denmark; ^17^ Laboratory of Polar Biology and Oceanobiology, Faculty of Biology and Environmental Protection University of Łódź Łódź Poland; ^18^ German Centre for Integrative Biodiversity Research (iDiv) Halle‐Jena‐Leipzig Leipzig Germany; ^19^ Department of Biological Sciences Bridgewater State University Bridgewater Massachusetts; ^20^ School of Biological Sciences The University of Hong Kong Pok Fu Lam Hong Kong; ^21^ CSIRO Land & Water Ecosciences Precinct, Dutton Park Queensland Australia; ^22^ Marine Science Institute, University of California Santa Barbara California; ^23^ Department of Biology University of New Mexico Albuquerque New Mexico; ^24^ Institute of Biology/Geobotany and Botanical Garden, Martin‐Luther‐University Halle‐Wittenberg Halle Germany; ^25^ Department of Watershed Sciences and the Ecology Center, US Geological Survey, UCFWRU and Utah State University Logan Utah; ^26^ Universidade do Extremo Sul Catarinense (PPG‐CA) Criciúma Santa Catarina Brazil; ^27^ Southeast Environmental Research Center (OE 148), Florida International University Miami Florida; ^28^ Coral Reef Ecology and Evolution Lab Biodiversity Research Centre, Academia Sinica Taipei Taiwan; ^29^ Anthropology, University of Georgia Athens Georgia; ^30^ Marine Biology and Aquaculture, College of Science and Engineering James Cook University Douglas Queensland Australia; ^31^ Memorial University, St John's Newfoundland and Labrador Canada; ^32^ Environmental Studies Program University of Colorado-Boulder; ^33^ Center for Tropical Forest Science Washington District of Columbia; ^34^ Biosciences Fisheries and Economics UiT‐ The Arctic University of Norway Tromsø Norway; ^35^ Systems Ecology, Department of Ecological Science, Vrije Universiteit Amsterdam The Netherlands; ^36^ AZTI Fundazioa, Herrera Kaia Pasaia Spain; ^37^ The National Institute of Water and Atmospheric Research Auckland New Zealand; ^38^ Lab of Vegetation Ecology, Instituto de Biociências, Universidade Estadual Paulista (UNESP), Rio Claro Brazil; ^39^ CSIRO Oceans and Atmosphere Flagship Hobart Tasmania Australia; ^40^ School of Science Edith Cowan University Joondalup Western Australia Australia; ^41^ Department of Biological Sciences Old Dominion University Norfolk Virginia; ^42^ Royal Belgian Institute of Natural Sciences, Operational Directorate Natural Environment, Marine Ecology and Management Brussels Belgium; ^43^ Marine Biology Research Group, Ghent University Gent Belgium; ^44^ School of Life and Environmental Sciences Centre for Integrative Ecology (Burwood Campus), Deakin University Geelong Victoria Australia; ^45^ Joint Nature Conservation Committee Aberdeen United Kingdom; ^46^ Divisão de Florestas e Estações Experimentais, Floresta Estadual de Assis, Laboratório de Ecologia e Hidrologia Florestal, Instituto Florestal São Paulo Brazil; ^47^ Tennenbaum Marine Observatories Network, Smithsonian Institution Washington, District of Columbia; ^48^ National Institute of Oceanography, Tel‐Shikmona Haifa Israel; ^49^ Institute for Marine and Antarctic Studies, University of Tasmania Hobart Tasmania Australia; ^50^ Hopkins Marine Station, Stanford University, Stanford California; ^51^ Department of Biological and Environmental Sciences University of Gothenburg Gothenburg Sweden; ^52^ Department of Wildlife Ecology and Conservation University of Florida Gainesville FL; ^53^ Instituto Milenio de Oceanografía, Universidad de Concepción Concepción Chile; ^54^ CSIC, Global Ecology Unit CREAF‐CSIC‐UAB Bellaterra Catalonia Spain; ^55^ CREAF, Universitat Autònoma de Barcelona Cerdanyola del Vallès Catalonia Spain; ^56^ Migratory Bird Center, Smithsonian Conservation Biology Institute, National Zoological Park Washington District of Columbia; ^57^ National Museum of Marine Biology and Aquarium Pingtung County Taiwan; ^58^ Laboratório de Ecologia e Restauração Florestal, Escola Superior de Agricultura “Luiz de Queiroz”, Universidade de São Paulo São Paulo Brazil; ^59^ Departamento de Oceanografia e Ecologia, Universidade Federal do Espírito Santo, Vitória, Espírito Santo Brazil; ^60^ Centre for Ecology, Evolution and Environmental Changes – cE3c, Faculty of Sciences University of Lisbon Lisbon Portugal; ^61^ Biological Dynamics of Forest Fragments Project, National Institute for Amazonian Research and Smithsonian Tropical Research Institute Manaus Brazil; ^62^ Department of Ecology/PPGE Federal University of Rio de Janeiro Rio de Janeiro Brazil; ^63^ School of Biological Sciences University of Aberdeen Aberdeen United Kingdom; ^64^ Botanical Department, Faroese Museum of Natural History Torshavn Faroe Islands; ^65^ Instituto Florestal, Seção de Ecologia Florestal São Paulo Brazil; ^66^ Polar Oceans Research Group Sheridan Montana; ^67^ Alexander von Humboldt Biological Resources Research Institute Bogotá DC Colombia; ^68^ Department of Biology Tomsk State University Tomsk, Russia; ^69^ USDA Forest Service, 65 USDA Forest Service, International Institute of Tropical Forestry San Juan Puerto Rico; ^70^ Center for Limnology, University of Wisconsin Madison Wisconsin; ^71^ The Warnell School of Forestry and Natural Resources University of Georgia Athens Georgia; ^72^ Departamento de Biología, Facultad de Ciencias, Universidad de La Serena La Serena Chile; ^73^ Centro de Estudios Avanzados en Zonas Aridas (CEAZA) La Serena Chile; ^74^ Institute of Ecology and Biodiversity (IEB) Santiago Chile; ^75^ U.S. Environmental Protection Agency, Office of Research and Development, National Health and Environmental Effects Research Laboratory, Atlantic Ecology Division Narragansett Rhode Island; ^76^ Department of Forest Ecosystems and Society Oregon State University Corvallis Oregon; ^77^ The Energy and Resources Group and The Department of Environmental Science, Policy and Management University of California Berkeley California; ^78^ Cetacean Research & Rescue Unit Banff United Kingdom; ^79^ U.S. Forest Service Pacific Northwest Research Laboratory Corvallis Oregon; ^80^ Laboratório de Dendrologia e Fitossociologia, Universidade do Estado de Santa Catarina Florianópolis Santa Catarina Brazil; ^81^ Department of Aquatic Environment and Quality, Flanders Research Institute for Agriculture, Fisheries and Food Oostende Belgium; ^82^ Norwegian Institute for Nature Research Trondheim Norway; ^83^ Department of Natural Resources and Cornell Biological Field Station Cornell University Ithaca New York; ^84^ Biology Department Grand Valley State University Allendale Michigan; ^85^ Dartmouth College Hanover New Hampshire; ^86^ Institute of Oceanography, National Taiwan University Taipei Taiwan; ^87^ University of California, Los Angeles Los Angeles California; ^88^ EWHALE lab‐ Biology and Wildlife Department Institute of Arctic Biology, University of Alaska Fairbanks Alaska; ^89^ Monterey Bay Aquarium Research Institute Moss Landing California; ^90^ Department of Biology University of North Carolina Chapel Hill North Carolina; ^91^ Institute of Marine Research His Norway; ^92^ Department of Biology University of Saskatchewan Saskatoon Saskatchewan Canada; ^93^ College of Earth, Ocean, and Atmospheric Sciences, Oregon State University Corvallis Oregon; ^94^ Wildlife Conservation Society Indonesia Program Bogor Indonesia; ^95^ Department of Wildlife Fish, and Conservation Biology, University of California, Davis Davis California; ^96^ Shetland Oil Terminal Environmental Advisory Group (SOTEAG) St Andrews United Kingdom; ^97^ Faculty of Environmental Sciences and Natural Resource Management Norwegian University of Life Sciences Ås Norway; ^98^ Department of Natural Sciences, Faculty of Engineering and Science, Centre for Coastal Research, University of Agder Kristiansand Norway; ^99^ Florida State University Coastal and Marine Laboratory St Teresa Florida; ^100^ Channel Islands National Park, U. S. National Park Service California, Ventura California; ^101^ Zoology, Ryan Institute, School of Natural Sciences, NUI Galway Galway Ireland; ^102^ Forest and Climate Change Adaptation Laboratory Center for Forest and Climate Change, National Institute of Forest Science Seoul Republic of Korea; ^103^ Department of Biological Sciences Virginia Institute of Marine Science, The College of William & Mary, Gloucester Point Virginia; ^104^ Département des sciences de l'environnement Université du Québec à Trois‐Rivières and Centre d’études nordiques Québec Canada; ^105^ Department of Biology Museum of Southwestern Biology, University of New Mexico Albuquerque New Mexico; ^106^ Vermont Center for Ecostudies Hartford Vermont USA; ^107^ Museu de Ciències Naturals de Granollers Catalunya Spain; ^108^ Hawai‘i Institute of Marine Biology, University of Hawai‘i at Mānoa, Kaneohe Hawai‘i USA; ^109^ Department of Biological Sciences Macquarie University Sydney New South Wales Australia; ^110^ Icelandic Institute of Natural History Garðabær Iceland; ^111^ School of Biology and Ecology Sustainability Solutions Initiative, University of Maine Orono Maine; ^112^ INSTAAR, University of Colorado Boulder Colorado; ^113^ Stream Biomonitoring Program, Mainspring Conservation Trust Franklin North Carolina; ^114^ Department of Biological Sciences University of Idaho Moscow Idaho; ^115^ Ecosystems and Environment Research Centre (EERC), School of Environment and Life Sciences, University of Salford Salford United Kingdom; ^116^ Terrestrial Ecology Section, Department of Biology, University of Copenhagen Copenhagen Denmark; ^117^ Laboratory of Phytoresources, Kovalevsky Institute of Marine Biological Research of RAS (IMBR) Sevastopol Russia; ^118^ Aquatic Survey & Monitoring Ltd. ASML Durham United Kingdom; ^119^ Ceiba Consultoria Ambiental Bragança Paulista Brazil; ^120^ Field Station Fabrikschleichach, University of Würzburg Rauhenebrach Germany; ^121^ Department of Biology Dalhousie University Halifax Nova Scotia Canada; ^122^ School of Geosciences University of Edinburgh Edinburgh United Kingdom; ^123^ Biology Department Oklahoma State University Oklahoma City Oklahoma; ^124^ Zoological Institute, Russian Academy Sciences St Petersburg Russia; ^125^ Marine Scotland, Marine Laboratory Scottish Government Edinburgh United Kingdom; ^126^ Department of Biology University of Louisiana at Lafayette Lafayette Louisiana; ^127^ BirdWatch Ireland Kilcoole Wicklow Ireland; ^128^ University of Arizona Health Sciences Library, University of Arizona Tucson Arizona; ^129^ Center for Forest Research, Université du Québec à Montréal (UQAM) Montreal Quebec Canada; ^130^ UMR AMAP, IRD, CIRAD, CNRS, INRA, Montpellier University Montpellier France; ^131^ Subtidal Ecology Laboratory & Center for Marine Conservation, Estación Costera de Investigaciones Marinas Facultad de Ciencias Biológicas, Pontificia Universidad Católica de Chile Santiago Casilla Chile; ^132^ Australian Centre of Field Robotics, University of Sydney Sydney New South Wales Australia; ^133^ Department of Aquatic Ecology Eawag: Swiss Federal Institute of Aquatic Science and Technology Switzerland; ^134^ Resource Ecology Group, Wageningen University Wageningen The Netherlands; ^135^ Department of Land Resources and Environmental Sciences Montana State University Bozeman Montana; ^136^ Entomology, University of Arizona Tucson Arizona; ^137^ Cold Spring Harbor Laboratory Cold Spring Harbor New York; ^138^ Ichthyology Laboratory, Fisheries and Aquaculture University of Aveiro Aveiro Portugal; ^139^ Department of Forest Resources, University of Minnesota St Paul Minnesota; ^140^ Hawkesbury Institute for the Environment, Western Sydney University Penrith New South Wales Australia; ^141^ CSIRO Oceans and Atmosphere Queensland, BioSciences Precinct (QBP) St Lucia, Brisbane Qld Australia; ^142^ Centre for Applications in Natural Resource Mathematics, The University of Queensland St Lucia Queensland Australia; ^143^ Virginia Institute of Marine Science Gloucester Point Virginia; ^144^ Metapopulation Research Centre, Faculty of Biosciences, University of Helsinki Helsinki Finland; ^145^ Department of Oceanography and Coastal Sciences, College of the Coast and Environment Louisiana State University Baton Rouge Louisiana; ^146^ Swiss Federal Institute for Forest, Snow and Landscape Research Davos Dorf Switzerland; ^147^ Departamento de Zoologia e Botânica, Universidade Estadual Paulista – UNESP Câmpus São José do Rio Preto, São José do Rio Preto Brazil; ^148^ Department of Animal Physiology, Eberhard Karls University Tübingen Tübingen Germany; ^149^ National Research Program, U.S. Geological Survey Reston Virginia; ^150^ Wisconsin Department of Natural Resources and Center for Limnology University of Wisconsin‐Madison Madison Wisconsin; ^151^ Department of Life Sciences Imperial College London Ascot Berkshire United Kingdom; ^152^ Departamento de Biologia Vegetal, UNICAMP Campinas Brazil; ^153^ Departamento de Ciências Biológicas, Escola Superior de Agricultura ‘Luiz de Queiroz’, Universidade de São Paulo São Paulo Brazil; ^154^ Department of Biology Utica College Utica New York; ^155^ Wildlife Ecology and Conservation, Department of Natural Resources and Environmental Sciences University of Illinois Champaign Illinois; ^156^ Biodiversity Research Center, Academia Sinica Nankang, Taipei Taiwan; ^157^ Marine Biological Laboratory, Woods Hole Massachusetts USA; ^158^ Maine Department of Marine Resources Bangor Maine; ^159^ Tulane University New Orleans Louisiana; ^160^ Environmental Sciences Department Federal University of São Carlos Sorocaba Brazil; ^161^ USP/WSAOBIS São Paulo Brazil; ^162^ Department of Biological Sciences, Centre for Statistics in Ecology, Environment and Conservation University of CapeTown Rondebosch South Africa; ^163^ Fynbos Node, South African Environmental Observation Network Claremont South Africa; ^164^ Coastal Finfish Section, South Carolina Department of Natural Resources, Marine Resources Research Institute Charleston South Carolina; ^165^ Conservation Biology Department Institute of Environmental Studies, CML, Leiden University Leiden The Netherlands; ^166^ Laboratório de Biologia e Tecnologia Pesqueira, Universidade Federal do Rio de Janeiro Rio de Janeiro Brazil; ^167^ Instituto Florestal, Seção de Ecologia Florestal São Paulo Brazil; ^168^ College of Marine Science University of South Florida St. Petersburg Florida; ^169^ Ethica Ambiental Vila Velha Brazil; ^170^ INBO, Research Institute for Nature and Forest Brussels Belgium; ^171^ Centro de Estudos em Recursos Naturais, Universidade Estadual de Mato Grosso do Sul Dourados Mato Grosso do Sul Brazil; ^172^ School of Biology and Ecology University of Maine Orono Maine; ^173^ Natural Resources Institute Finland, University of Oulu Oulu Finland; ^174^ Instituto Florestal, Divisão de Florestas e Estações Experimentais, Estação Experimental de Bauru Bauru Brazil; ^175^ Department of Biology University of Bergen Bergen Norway; ^176^ GEOMATRIX UAB Kaunas Lithuania; ^177^ Universidad Austral de Chile and Centro FONDAP en Dinámica de Ecosistemas Marinos de Altas Latitudes (IDEAL) Valdivia Chile; ^178^ Department of Biology Saint Louis University Saint Louis Missouri; ^179^ Escola de Agronomia, Universidade Federal de Goiás Goiânia Brazil; ^180^ Department of Environmental Affairs Oceans and Coastal Research Cape Town South Africa; ^181^ Department of Biological Sciences Marine Research Institute University of Cape Town Cape Town South Africa; ^182^ Institute of Ecology and Biological Resources, VAST Hanoi Vietnam; ^183^ Vietnam National Museum of Nature Hanoi Vietnam; ^184^ Graduate University of Science and Technology, VAST Hanoi Vietnam; ^185^ Biology Department, Drew University Madison New Jersey; ^186^ Environmental Studies Department, Drew University Madison New Jersey; ^187^ Laboratory of Forest Biology Wrocław University Wroclaw Poland; ^188^ Department of Wildlife Ecology & Conservation University of Florida Gainesville Florida; ^189^ Informatics Institute, University of Florida Gainesville Florida; ^190^ Plymouth Marine Laboratory Plymouth United Kingdom; ^191^ Department of Natural Sciences McPherson College McPherson Kansas; ^192^ Australian Antarctic Division, Channel Highway Kingston Tasmania Australia; ^193^ Department of Biology University of York York United Kingdom; ^194^ Department of Ecology & Evolutionary Biology, Center for Environmental Sciences & Engineering University of Connecticut Mansfield Connecticut; ^195^ Institute of Marine Sciences, School of Biological Sciences, University of Portsmouth Portsmouth United Kingdom; ^196^ Research Team Mountain Ecosystems, WSL Institute for Snow and Avalanche Research SLF Davos Switzerland; ^197^ Natural Sciences, Bennington College Bennington Vermont; ^198^ Leibniz Institute for Baltic Sea Research Warnemünde, Seestr. 15, D‐18119 Rostock Germany

**Keywords:** biodiversity, global, spatial, species richness, temporal, turnover

## Abstract

**Motivation:**

The BioTIME database contains raw data on species identities and abundances in ecological assemblages through time. These data enable users to calculate temporal trends in biodiversity within and amongst assemblages using a broad range of metrics. BioTIME is being developed as a community‐led open‐source database of biodiversity time series. Our goal is to accelerate and facilitate quantitative analysis of temporal patterns of biodiversity in the Anthropocene.

**Main types of variables included:**

The database contains 8,777,413 species abundance records, from assemblages consistently sampled for a minimum of 2 years, which need not necessarily be consecutive. In addition, the database contains metadata relating to sampling methodology and contextual information about each record.

**Spatial location and grain:**

BioTIME is a global database of 547,161 unique sampling locations spanning the marine, freshwater and terrestrial realms. Grain size varies across datasets from 0.0000000158 km^2^ (158 cm^2^) to 100 km^2^ (1,000,000,000,000 cm^2^).

**Time period and grain:**

BioTIME records span from 1874 to 2016. The minimal temporal grain across all datasets in BioTIME is a year.

**Major taxa and level of measurement:**

BioTIME includes data from 44,440 species across the plant and animal kingdoms, ranging from plants, plankton and terrestrial invertebrates to small and large vertebrates.

**Software format:**

.csv and .SQL.

## 1 BACKGROUND

Quantifying changes in biodiversity in the Anthropocene is a key challenge of our time given the paucity of temporal and spatial data for most taxa on Earth. The nature and extent of the reorganization of natural assemblages are currently controversial because conflicting estimates of biodiversity change have been obtained using different methodological approaches and for different regions, time periods and taxa. Some reports suggest alarming and systematic biodiversity loss. For example, estimates of global extinction rates place global losses orders of magnitude above background rates (Pereira, Navarro, & Martins, 2012). In addition, estimates of population trends for vertebrates suggest average declines of the order of 60% in the past 30 years (Collen et al., 2009). Nonetheless, analyses based on spatial variation yield more modest declines in the range of 8% (Newbold et al., 2015). In contrast, some analyses of assemblage time series consistently detect no systematic trend in temporal α‐diversity (such as species richness), on average, across local communities (Brown, Ernest, Parody, & Haskell, 2001; Dornelas et al., 2014; Vellend et al., 2013, 2016), but instead uncover substantial variation in composition (temporal β‐diversity; i.e., temporal turnover), including both losses and gains of species (Dornelas et al., 2014; Magurran, Dornelas, Moyes, Gotelli, & McGill, 2015). Spatially structured gains and losses are also predicted from climate change projections (García Molinos et al., 2016). Some of these discrepancies are a result of differences in the temporal and spatial scales at which analyses were performed (McGill, Dornelas, Gotelli, & Magurran, 2014), whereas other differences may be attributable to the organizational level on which an analysis is focused (e.g., population vs. community). Clearly, more research is needed into how populations, communities and ecosystems are changing in the face of widespread human influence on the planet (Waters et al., 2016). Here, we introduce BioTIME, a curated database of biodiversity time series, with the goal of facilitating and promoting research in this area.

Biodiversity is a multifaceted concept, which can be measured in many different ways. Similar to the approach of essential biodiversity variables (Pereira et al., 2013), we focus on assembling data that maximize the number of metrics that can be calculated. Specifically, BioTIME is composed of species abundance records for assemblages that have been sampled through time with a consistent methodology. The focus on assemblages differentiates BioTIME from population databases, such as the Global Population Dynamics Database (https://www.imperial.ac.uk/cpb/gpdd2/secure/login.aspx) and the Living Planet Index database (http://www.livingplanetindex.org/home/index), and enables users to quantify patterns at different organizational levels, including both the assemblage and the population level. BioTIME complements the PREDICTS database (http://www.predicts.org.uk/) in providing time series rather than space for time comparisons. Moreover, most previous databases have been either terrestrial (e.g., vertebrates, GPDD; vegetation, sPlot; multiple taxa, PREDICTS) or marine (e.g., OBIS), whereas BioTIME includes marine, freshwater and terrestrial realms; hence, it facilitates comparisons across realms. Finally, previous databases are not specifically focused on temporal assemblage data, which means that BioTIME fills an important gap in allowing spatial and temporal comparisons. In addition, coupling BioTIME with additional information will allow analyses of temporal change in phylogenetic diversity and trait diversity alongside taxonomic diversity.

The goals of the BioTIME database are as follows: (a) to assemble and format raw species abundance data for assemblages consistently sampled through time; (b) to encourage re‐use of these data through open‐source access of standardized and curated versions of the data; and (c) to promote appropriate crediting of data sources. These goals are in line with best practice in promoting maximal use of ecological data (Costello et al., 2014; White et al., 2013) and highlight data gaps to funding agencies. In addition, we hope that BioTIME will engage ecologists in the collection, standardization, sharing and quality control of assemblage‐level species abundance data, particularly in poorly sampled parts of the world, and highlight the value of such data to funding agencies.

## 2 METHODS

The BioTIME database is composed of 11 tables: a main table containing the core observations (records), and 10 tables that provide contextual information as described below and in Supporting Information Figure S1. There are five main levels of organization: record, sample, plot, site and study. A record is our fundamental unit of observation of the abundance of a species in a sample. A sample includes all the records that belong to the same sampling event; for example, a quadrat on the seashore, a single plankton tow or a bird transect. A sample is defined by a single location and a single date. If the exact location has been repeatedly sampled through time, then all the samples that correspond to that location belong to the same plot. Multiple samples and plots can be located in the same area, which we term a site. Finally, the highest observational unit is a study, which is defined by having a regular and consistent sampling methodology. Sources of data in which the sampling methodology changed during the course of the study were classified as separate studies. Every organizational level has contextual variables that are kept either in dedicated tables or are part of the main table (see Supporting Information Figure S1 for a complete list of the fields in each table). In addition, the database also includes tables with information relating to the sampling methodology, and treatments associated with some samples when applicable, citation information, contacts and licenses for each study, and the curation steps performed on each study before it was entered in the database.

### 2.1 Data acquisition

Searches began in 2010, and data were acquired from a variety of sources: literature searches, large databases [specifically, OBIS (http://www.iobis.org/), GBIF (http://www.gbif.org/) and Ecological Data Wiki (https://ecologicaldata.org/)], through personal networking and through broadcasted data requests at conferences and on social media. We have used four main criteria for data inclusion on BioTIME: (a) abundance observations come from samples of assemblages where all individuals within the sample were counted and identified (i.e., assemblage rather than population data); (b) most of the individuals were identified to species; (c) sampling methods were constant through time; and (d) the time series spans a minimum of 2 years. The last condition was changed relative to the initial criteria because it became apparent that it would allow better spatial representation given the many locations that have been surveyed historically and then resurveyed. Each study is kept separate within the database and has a specific license from the CC spectrum, whose terms must be observed (https://creativecommons.org/). A static version of the database is released with this publication (http://biotime.st-andrews.ac.uk and https://zenodo.org/record/1095627). However, data entry and curation is ongoing (http://biotime.st-andrews.ac.uk/contribute.php), and we expect the database to keep growing in the foreseeable future. We plan to release static updates of the database periodically.

### 2.2 Data curation and quality control

Before inclusion in the database, data were subjected to standardization in a curation process described specifically for each study in the curation table of the database. Specifically, these were checked for the presence of the following: duplicates within each study and against the entire database; species with zero abundance; and non‐organismal records, all of which were removed. Abundances of zero for a particular population can be inferred from their absence from samples in the study. Additionally, species names were checked for typographic errors and misspellings, and a standardized notation was used for records of morphospecies and species complexes. Most records were included as provided and may not always conform to the latest nomenclature. Furthermore, latitudes and longitudes were checked for their location relative to other descriptors (e.g., country or marine vs. terrestrial). Finally, the grain and extent of each study were calculated from information in the methods where available, or by applying a convex hull algorithm to locations of the samples.

## 3 DESCRIPTION OF DATA

**Figure 1:**
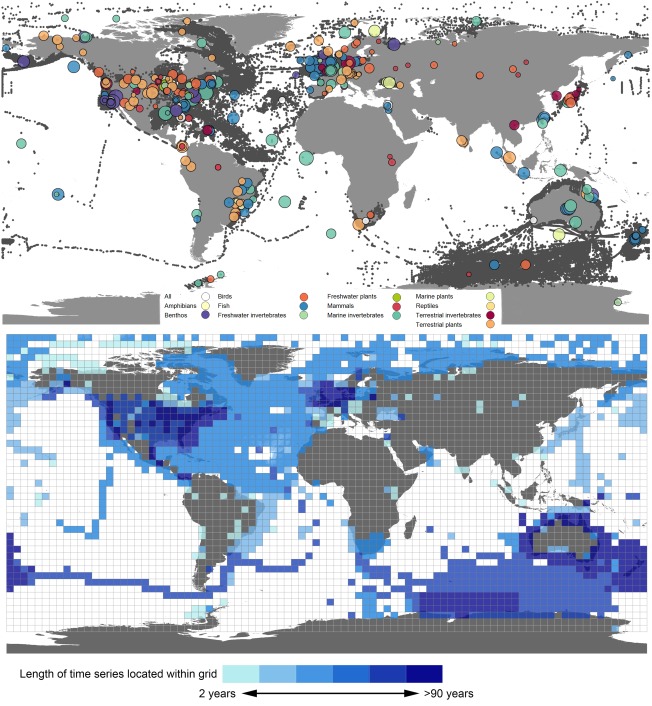
Top: Geographical locations of all the records included in BioTIME in dark grey, with central points per study shown as circles of different colour and size, according to taxa and number of species. Bottom: Map overlaid with ∼4° grid cells coloured by the length of the full or partial time series contained within each cell

In total, the version of BioTIME released with this paper includes 8,777,413 records, across 547,161 unique locations, gathered from 361 studies (Figure 1; see Appendix for a full list of citations). These observations span the Poles to the Equator, from depths of *c*. 5,000 m to elevations of *c*. 4,000 m above sea level, and include the terrestrial, freshwater and marine realms. The database includes records spanning 21 out of 26 ecoregions [WWF; (http://www.worldwildlife.org/biomes)]. Nonetheless, there are spatial biases in the distribution of sampling locations, with most studies occurring in Europe, North America and Australia. This geographical bias has persisted despite the growth of the database. For example, a comparison between Supporting Information Figure S2 and the data included in the study by Dornelas et al. (2014) displays only small differences, despite the database having more than tripled its size in the interim. It is our hope that this geographical bias will decrease over time via targeted searches and data recruitment.

**Figure 2:**
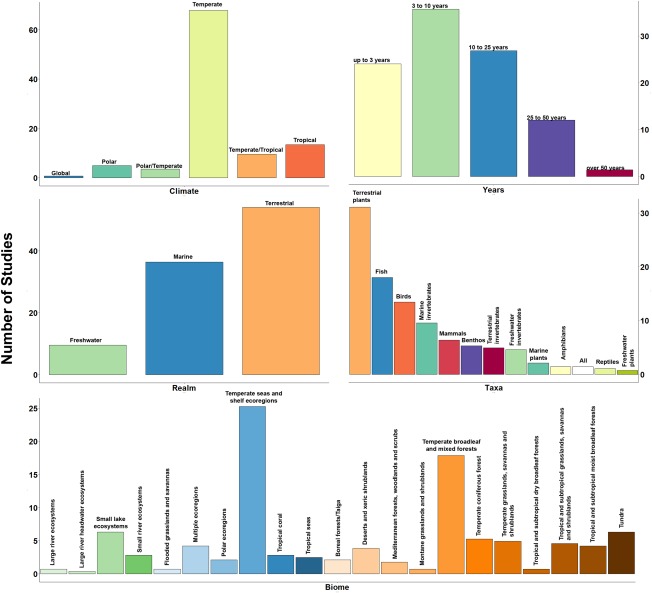
Proportion of studies that fall into the different classifications of: Climate, number of years sampled, realm, taxa and biome

There are 44,440 taxa in BioTIME. The majority of these (88.8%) are species, but some organisms are identified only to coarser taxonomic levels, such as genus. BioTIME includes assemblages across the animal and plant kingdoms, ranging from mammals to microscopic plankton. As with the spatial distribution, there are also taxonomic biases in the data in BioTIME (Figure 2). Almost 70% of records fall into one of four categories: terrestrial plants, birds, fish and marine invertebrates, with fish accounting for 28% of the total database.

BioTIME records span 118 years (from 1874 to 2016), with the longest time series having 97 years and an average duration of 13 years. In more detail, 56.5% of studies contain up to 10 years of data, 42% between 10 and 50 years and 1.4% > 50 years.

## 4 USAGE NOTES

Version 1.0 of the BioTIME database can be downloaded from https://zenodo.org/record/1095627 or from http://biotime.st-andrews.ac.uk/. The use of data contained in BioTIME should cite original data citations in addition to the present paper. There is considerable variation in the spatial and temporal grain and extent among studies, which must be considered in any analysis of BioTIME data. Moreover, the number of samples was often not constant through time within studies; consequently, we recommend the use of sample‐based rarefaction and provide R code to query the database, implement sample‐based rarefaction and calculate a suite of biodiversity metrics. Specifically, we provide a tutorial guiding users to interact with both formats of the database (.csv and .sql; Allaire et al., 2015; Becker, Wilks, & Brownrigg, 2014; Oksanen et al., 2013; Ooms, James, DebRoy, Wickham, & Horner, 2015; R Development Core Team, 2013; Wickham, 2009; Wickham & Francois, 2015). Please note that for interacting with the .sql version of the database, users will have to set up a connection with the server where they have installed the SQL database. For interacting with the .csv version, users have to download both the data and the metadata csv files, making sure that all the paths to these files are modified accordingly.

The data included in the present paper represent the subset of data within the BioTIME database for which we were able to secure licences to republish. The additional studies held in the full database have been obtained from publicly available data and are listed in Supporting Information Table S1. In total, BioTIME currently holds 387 studies, containing 12,623,386 records from a total of 652,675 distinct geographical locations, and 45,093 species. These records span a total of 124 years from 1858 to 2016 inclusive. We will continue to interact with data providers in order to increase data availability and to recruit additional data. Instructions on how to contribute to future releases can be found here (http://biotime.st-andrews.ac.uk/contribute.php).

## DATA ACCESSIBILITY

The BioTIME database is accessible through the BioTIME website (http://biotime.st-andrews.ac.uk) and through the Zenodo repository (https://zenodo.org/record/1095627).

BIOSKETCH The **BioTIME consortium** emerged from the ERC project BioTIME in 2010. The consortium currently includes 271 authors distributed among 35 countries engaged in collecting biodiversity time series data and committed to sharing it for wider use. We hope that the BioTIME database allows analysis of large‐scale patterns of biodiversity change and contributes to giving credit to the data collectors, without whom synthesis would not be possible.

## Supporting information

Additional Supporting Information may be found online in the supporting information tab for this article.

Supporting Information

Supporting Information

Supporting Information
