# Supplementary material for: BioTIME: A database of biodiversity time series for the Anthropocene
Source: Glob Ecol Biogeogr. 2018 Jul 24;27(7):760–86. doi: 10.1111/geb.12729 (PMC6099392; doi:10.1111/geb.12729)
Supplement: Supplementary file 1 — Supporting Information [file GEB-27-760-s001.pdf]

Interacting with the BioTIME database

BioTIME consortium

22/06/2017

This tutorial guides users interacting with BioTIME, the largest compilation of biodiversity time series containing raw data on species identities and abundances from ecological assemblages (Dornelas *et al*). The database can be accessed through [ZENODO] “to insert link later” and the BioTIME website (https://www.st-andrews.ac.uk/biotime/bioTIME/download.php).

We provide code and examples to calculate simple summary statistics describing the data, and to produce descriptive plots and maps.

The BioTIME database

BioTIME is a relational database built using the mySQL language, and contains eleven linked tables as illustrated in Fig. 1. Within each table, columns contain the field names, whilst rows contain the records or data for each column.

Each record has a unique identifier or primary key, and queries written in SQL (Structured Query Language) are used to communicate with the database.

BioTIME is available in two formats:

1. **MySQL database (.sql) file**
2. **Comma delimited (.csv) files** consisting of:
    - full query by study taken from the raw data table within the database
    - associated metadata for each dataset
    - full list of citations

Below we provide instructions and code for both formats. First, we show how to explore the database using MySQL language, and how to produce several outputs using the .sql file. It is possible to query the databse in order to produce downloadable csv files, containing data from the several tables. However, please note that the query that creates the rawdata csv file can take several hours to run, so we provide the file for download and you can skip to using the csv files.

Code to generate the query used in the second part of this document - for use after connection is made

```
fullQuery<-dbGetQuery(myConnection, "SELECT allrawdata.STUDY_ID, allrawdata.DAY, allrawdata.MONTH, allrawdata.YEAR, allrawdata.SAMPLE_DESC, allrawdata.PLOT, allrawdata.ID_SPECIES, allrawdata.LATITUDE,
allrawdata.LONGITUDE, sum(allrawdata.ABUNDANCE), sum(allrawdata.BIOMASS), species.GENUS, species.SPECIES, species.GENUS_SPECIES from allrawdata inner join site on allrawdata.STUDY_ID=site.STUDY_ID inner join
species on allrawdata.ID_SPECIES=species.ID_SPECIES group by concat(allrawdata.STUDY_ID, allrawdata.DAY, allrawdata.MONTH, allrawdata.YEAR, allrawdata.SAMPLE_DESC, allrawdata.ID_SPECIES, allrawdata.LATITUDE,
allrawdata.LONGITUDE)")
```

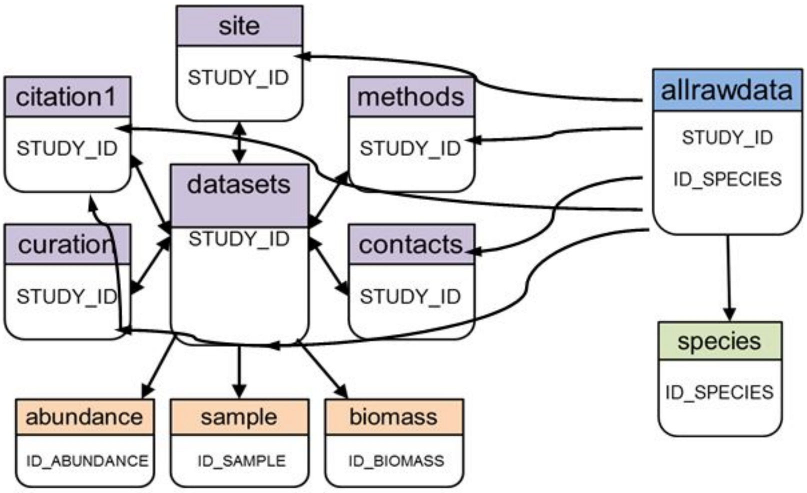

Figure 1 - BioTIME database structure, showing the linking field between the tables; for a full list of the fields and their description see Supplementary Materials in Dornelas et al.

Most of the tables in BioTIME are linked by the STUDY_ID field; the only exceptions to this are the links between *allrawdata* and *species* tables (ID_SPECIES), and the relationships between the *abundance* and *biomass* tables and *datasets* (ID_ABUNDANCE and ID_BIOMASS, respectively). This means that effectively *allrawdata* links to all the other tables with the exception of *abundance*, *biomass* and *sample*, which can be joined through the *datasets* table.

1.Using the MySQL database

The R package RMySQL allows a connection to any MySQL database using R (Studio). In order to use this, you must first download and extract the .sql file and import it to MySQL.

MySQL is open source, and is readily available for download and installation both as MySQL Server or through the installation of larger open source applications such as XAMPP.

Load the required libraries

```
library(RMySQL)
library(ggplot2)
library(mapdata)
library(vegan)
library(dplyr)
```

Create a connection to the database

```
myConnection <- dbConnect(MySQL(), user="username", host="hostname", dbname="database", password="password")
```

This section should be run as code once the appropriate names have been added.

Some simple queries to explore the database

```
### list the tables in the database
dbListTables(myConnection)
```

```
##  [1] "abundance"  "allrawdata" "biomass"    "citation1"  "contacts"
##  [6] "curation"   "datasets"   "methods"    "sample"     "site"
## [11] "species"
```

```
### or
dbGetQuery(myConnection, "show tables")
```

```
##    Tables_in_biopart
## 1          abundance
## 2         allrawdata
## 3            biomass
## 4          citation1
## 5           contacts
## 6           curation
## 7           datasets
## 8            methods
## 9             sample
## 10              site
## 11           species
```

```
### list the fields with their associated information in a chosen table
dbGetQuery(myConnection, "show columns from contacts")
```

```
##         Field         Type Null Key Default          Extra
## 1 ID_CONTACTS      int(11)   NO PRI    <NA> auto_increment
## 2    STUDY_ID      int(11)  YES MUL    <NA>
## 3   CONTACT_1 varchar(500)  YES        <NA>
## 4   CONTACT_2 varchar(500)  YES        <NA>
## 5 CONT_1_MAIL  varchar(60)   NO        <NA>
## 6 CONT_2_MAIL  varchar(60)  YES        <NA>
## 7     LICENSE varchar(200)   NO        <NA>
## 8    WEB_LINK varchar(200)  YES        <NA>
## 9 DATA_SOURCE varchar(250)   NO        <NA>
```

```
### get the list of fields from a chosen table
dbListFields(myConnection, "site")
```

```
## [1] "ID_SITE"        "STUDY_ID"       "REALM"          "CLIMATE"
## [5] "GENERAL_TREAT"  "TREATMENT"      "TREAT_COMMENTS" "TREAT_DATE"
## [9] "CEN_LATITUDE"   "CEN_LONGITUDE"  "HABITAT"        "PROTECTED_AREA"
## [13] "AREA"          "BIOME_MAP"
```

```
### read entire tables - N.B. not recommended unless the number of records is known and is not too large!
dbReadTable(myConnection, "biomass")
```

```
##   ID_BIOMASS     BIOMASS_TYPE
## 1          1               NA
## 2          2             Size
## 3          3           Weight
## 4          9 Relative biomass
## 5         10           Volume
## 6         12            Cover
```

```
dbReadTable(myConnection, "abundance")
```

```
##   ID_ABUNDANCE   ABUNDANCE_TYPE
## 1            1            Count
## 2            5 Presence/Absence
## 3            6        MeanCount
## 4            7          Density
## 5            8               NA
```

Build queries and wrap them using the dbGetQuery function

There are two ways to select the number of studies held in BioTIME

```
#### query the datasets table for the number of entries, as each record corresponds to each individual study
result<-dbGetQuery(myConnection, "SELECT count(*) FROM datasets")
```

```
## [1] "There are  359  studies in the database"
```

```
#### or interrogate the raw data table for the unique number of Study IDs
result2 <- dbGetQuery(myConnection, "SELECT count(distinct STUDY_ID) FROM allrawdata")
```

```
## [1] "There are  359  studies in the database"
```

Use an inner join to link the raw data table with the site table in order to count the number of species found in Polar regions, for instance

```
dbGetQuery(myConnection, "SELECT count(distinct ID_SPECIES) as PolarSpecies FROM allrawdata inner join site on allrawdata.ST
UDY_ID = site.STUDY_ID where site.CLIMATE = 'Polar'")
```

```
##   PolarSpecies
## 1         1258
```

The above queries were run using the count command to return a single numerical value, but queries can also be used to return blocks of data for analyses, for instance

```
dbGetQuery(myConnection, "SELECT distinct ID_SPECIES FROM allrawdata inner join site on allrawdata.STUDY_ID = site.STUDY_ID
 where site.CLIMATE = 'Polar'")
```

would return a list of all the species found in Polar regions, instead of the number of species.

Using query results to create maps and plots

```
#### select all the central latitudes and longitudes for each study, along with the taxonomic classification and total numbe
r of records
result<-dbGetQuery(myConnection, "SELECT CENT_LAT, CENT_LONG, TAXA, TOTAL from datasets")
```

Create the base map

```
#### draw a basic world map, add "y" or "n" for display of tropics and polar latitudes

drawWorld<-function(lats) {
  world_map<-map_data("world")

    g1<-ggplot()+coord_fixed()+xlab("")+ylab("")
    g1<-g1+geom_polygon(data=world_map, aes(x=long, y=lat, group=group), colour="gray60", fill="gray60")
    g1<-g1+theme(panel.grid.major=element_blank(), panel.grid.minor=element_blank(),
    panel.background=element_rect(fill="white", colour="white"), axis.line=element_line(colour="white"),
    legend.position="none",axis.ticks=element_blank(), axis.text.x=element_blank(), axis.text.y=element_blank())

    if(lats=="y") {
        g1<-g1+geom_hline(yintercept=23.5, colour="red")+geom_hline(yintercept =-23.5, colour="red")
        g1<-g1+geom_hline(yintercept=66.5, colour="darkblue")+geom_hline(yintercept =-66.5, colour="darkblue")
    }
    else { return(g1) }
        return(g1)
}
```

Build a colour palette for the taxa

```
taxaCol<-c('#ffffff','#ffffbf','#5e4fa2','#f46d43','#3288bd','#abdda4','#a8c614','#d53e4f','#66c2a5','#e6f598','#fee08b','#9
e0142','#fdae61')
```

Create world map with study central locations, with points coloured by taxa and sized by number of records

```
#### add the point coordinates to the blank map (without polar and tropic latitudinal lines)

points<-drawWorld("n")+geom_point(data=result, aes(x=CENT_LONG, y=CENT_LAT, colour=TAXA, size=TOTAL), alpha=I(0.7))
points<-points+scale_colour_manual(values= taxaCol)+scale_size(range=c(3, 10))
points
```

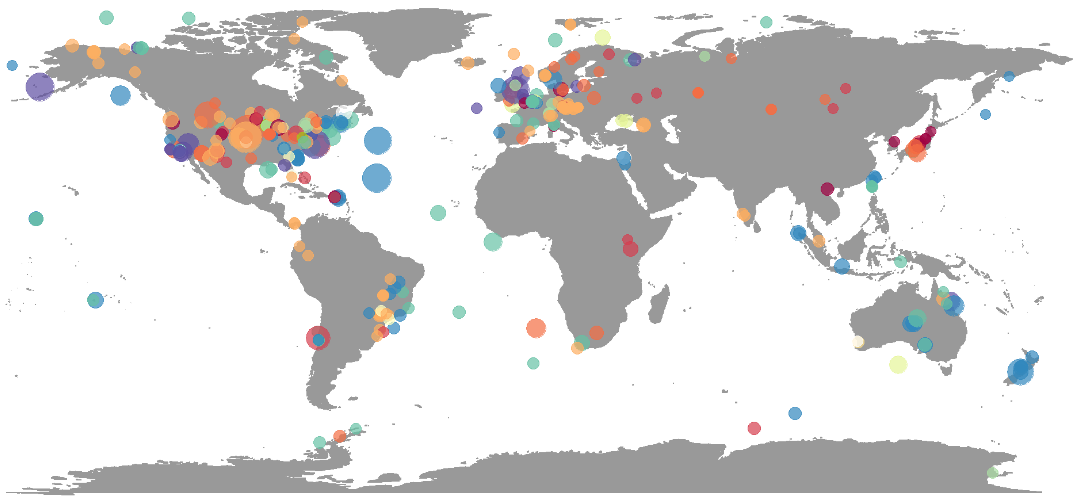

Basic theme for ggplots with no grid and no axes titles (useful for barplots)

```
#### theme no grid
themeNoGrid<-function() {
   theme_bw()+
        theme(axis.text=element_text(size=16,color="black"),
        axis.title=element_text(size=0,face="bold"),
        legend.position="none",
        plot.title=element_text(size=18,face="bold", hjust=0.5),
        plot.background=element_blank(),
        panel.grid.major=element_blank(),
        panel.grid.minor=element_blank(),
        panel.border=element_blank(),
        axis.line=element_line(color="black")
    )
}
```

Query the database to select the proportion of studies that fall into each taxonomic group

```
#### get studies by taxa
taxaFile<-dbGetQuery(myConnection, "SELECT distinct TAXA, ((count(TAXA)/359)*100) as valueX from datasets group by TAXA")
```

Function to create a barplot for the taxa, which takes variables for data, colour and position of y axis

```
taxaPlot<-function(x, cols, pos) {
    plot<-ggplot(x, aes(factor(TAXA), valueX, fill=TAXA))+
      geom_bar(stat="identity", width=0.93,position=position_dodge(width=1),colour="black")+
      scale_fill_manual(values= cols)+
      scale_y_continuous(position=pos)+themeNoGrid()+
      theme(axis.text.x=element_blank())+
      geom_text(aes(label=TAXA), vjust=-1, hjust=0.7, angle=0.5)
    return(plot)
}
```

```
taxaPlot(taxaFile, taxaCol, "left") ##axis is on the left
```

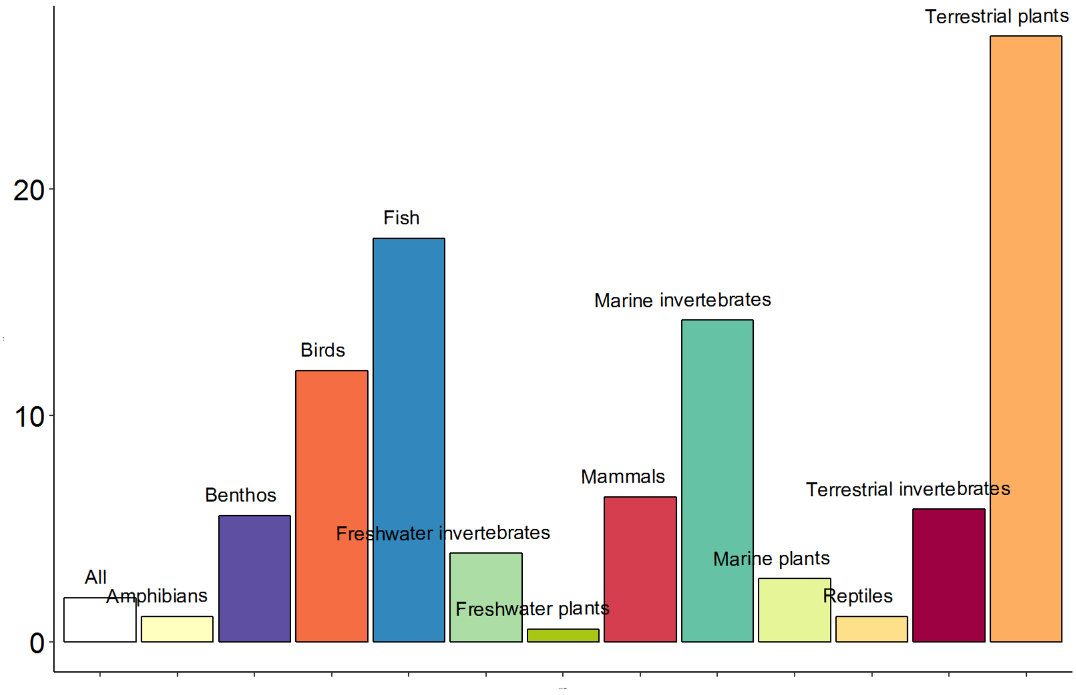

Create a similar plot using climate information, rather than taxa and omitting the record number variable

```
#### select studies by climate, use an inner join here to link the datasets and site tables
result<-dbGetQuery(myConnection, "SELECT datasets.CENT_LAT, datasets.CENT_LONG, site.CLIMATE FROM datasets inner join site o
n site.STUDY_ID = datasets.STUDY_ID")
```

Colour palette for the climate

```
###colours for the climate
climCol<-c('#3288bd','#66c2a5','#abdda4','#e6f598','#fdae61','#f46d43')
```

Create map with study central locations, coloured by climate and with the tropical and polar lines of latitude overlaid

```
#### add the point co-ordinates to the blank map (including polar and tropic latitudinal lines)

points<-drawWorld("y")+geom_point(data=result, aes(x=result$CENT_LONG, y=result$CENT_LAT, colour=result$CLIMATE), size=5, al
pha=I(0.7))
points<-points+scale_colour_manual(values= climCol)
points
```

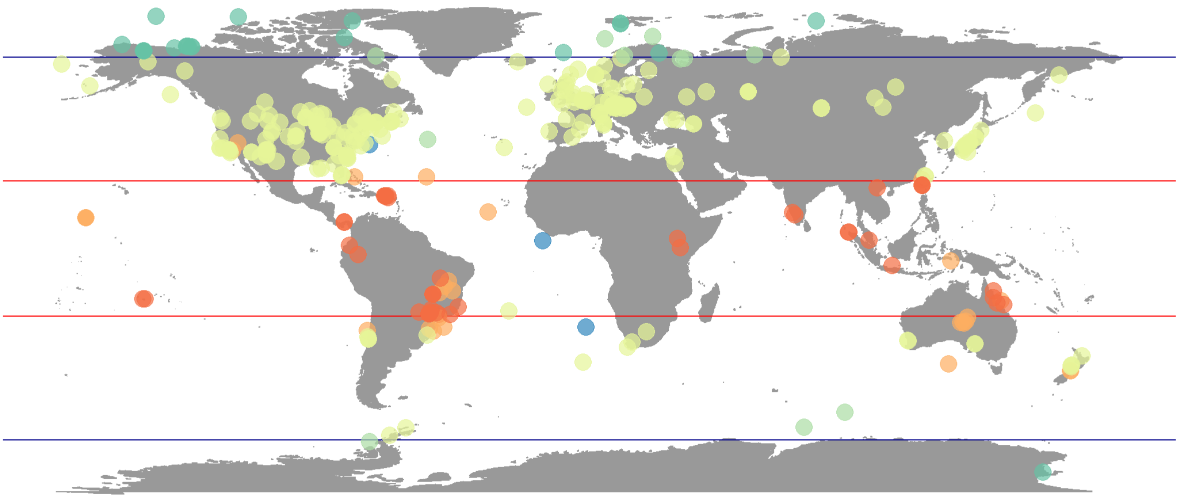

Function to create a barplot for the climate information (similar to taxa above)

```
climPlot<-function(x, cols, pos) {
    plot<-ggplot(x, aes(factor(CLIMATE), valueX, fill=CLIMATE))+
      geom_bar(stat="identity", width=0.93, position=position_dodge(width=1), colour="black")+
      scale_fill_manual(values= cols)+
      scale_y_continuous(position=pos)+
      themeNoGrid()+
      theme(axis.text.x=element_blank())+
      geom_text(aes(label=CLIMATE), vjust=-0.8, hjust=0.5, angle=0)
    return(plot)
}
```

Query the database to select the proportion of studies that fall into each climatic zone

```
#### get studies by climate
climFile<-dbGetQuery(myConnection, "SELECT distinct CLIMATE, ((count(CLIMATE)/359)*100) as valueX from site  group by CLIMAT
E")
```

```
climPlot(climFile, climCol, "right") ##axis is on the right
```

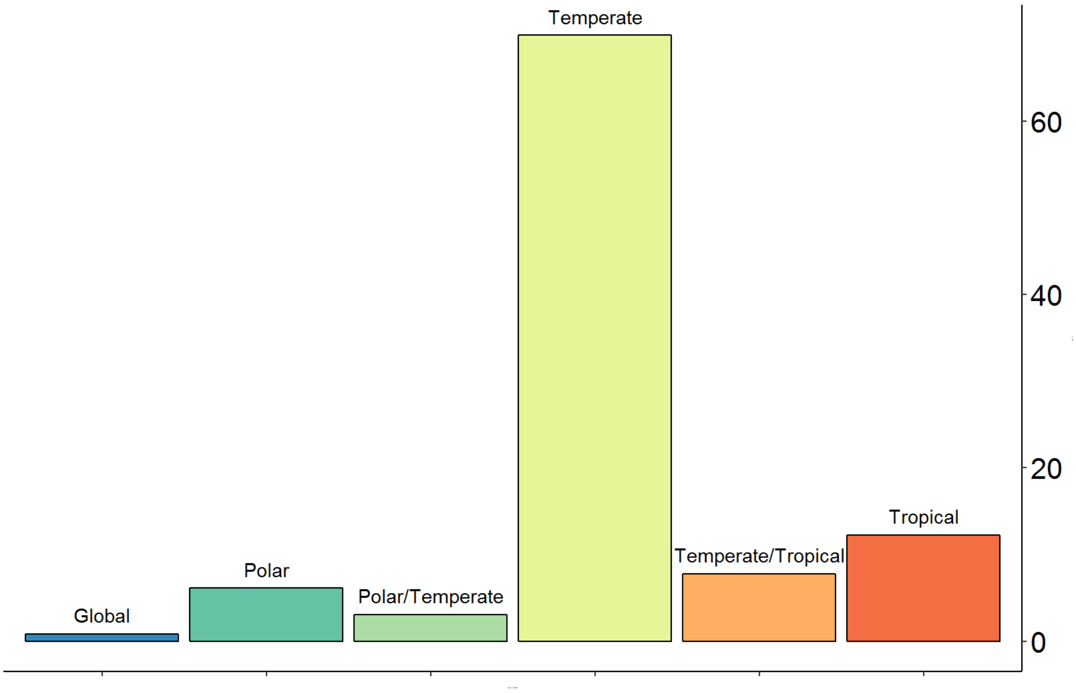

Some more examples of queries

Select the list of unique temperate mammal species. This requires information from four tables, as taxa is in *datasets*, climate in *site* and it is preferable to show names, rather than numerical IDs for the species

```
result<-dbGetQuery(myConnection, "SELECT distinct GENUS_SPECIES FROM allrawdata inner join site on site.STUDY_ID = allrawdat
a.STUDY_ID inner join datasets on datasets.STUDY_ID = allrawdata.STUDY_ID inner join species on species.ID_SPECIES = allrawd
ata.ID_SPECIES where datasets.TAXA='Mammals' and site.CLIMATE = 'Temperate'")

head(result)  #### showing only first entries
```

```
##                GENUS_SPECIES
## 1         Blarina transitans
## 2      Clethrionomys gapperi
## 3       Napaeozapus insignis
## 4              Peromyscus sp
## 5            Tamias striatus
## 6 Ammospermophilus interpres
```

Use the same four tables to select the number of unique tropical fish species

```
dbGetQuery(myConnection, "SELECT count(distinct GENUS_SPECIES) FROM allrawdata inner join site on site.STUDY_ID = allrawdat
a.STUDY_ID inner join datasets on datasets.STUDY_ID = allrawdata.STUDY_ID inner join species on species.ID_SPECIES = allrawd
ata.ID_SPECIES where datasets.TAXA='Fish' and site.CLIMATE = 'Tropical'")
```

```
##   count(distinct GENUS_SPECIES)
## 1                          1376
```

SQL uses the * key to select everything from a table (or group of tables), but it is also possible to select specific fields to be returned in the query result. In this example, the query will only export the abundance, latitude, longitude, year, and species fields from the study ID 302. By saving the query into an object ('result'), it can then be manipulated as a data frame in R or written out to a csv file.

```
result <- dbGetQuery(myConnection, "SELECT GENUS_SPECIES, allrawdata.ABUNDANCE, allrawdata.LATITUDE, allrawdata.LONGITUDE, a
llrawdata.YEAR FROM allrawdata inner join species on species.ID_SPECIES = allrawdata.ID_SPECIES where allrawdata.STUDY_ID=30
2")

head(result)
```

```
##                  GENUS_SPECIES ABUNDANCE LATITUDE LONGITUDE YEAR
## 1 Actinostemon  concepcionis\r         1  -22.379  -49.6803 2002
## 2 Actinostemon  concepcionis\r         1  -22.379  -49.6803 2002
## 3 Actinostemon  concepcionis\r         1  -22.379  -49.6803 2002
## 4 Actinostemon  concepcionis\r         1  -22.379  -49.6803 2002
## 5 Actinostemon  concepcionis\r         1  -22.379  -49.6803 2002
## 6 Actinostemon  concepcionis\r         2  -22.379  -49.6803 2002
```

```
write.csv(result, "result.csv") #### save the object as a csv file
```

Select the number of records where the study duration is greater than 50 years; this query only requires two tables - *allrawdata* and *datasets*.

```
dbGetQuery(myConnection, "SELECT count(*) FROM allrawdata inner join datasets on datasets.STUDY_ID = allrawdata.STUDY_ID whe
re datasets.DATA_POINTS>50")
```

```
##   count(*)
## 1   293311
```

or chose another condition, such as number of species

```
dbGetQuery(myConnection, "SELECT count(*) FROM allrawdata inner join datasets on datasets.STUDY_ID = allrawdata.STUDY_ID whe
re datasets.NUMBER_OF_SPECIES>100")
```

```
##   count(*)
## 1  7431420
```

There are a number of queries that do not need a join, as all necessary fields are within one table; for example, exporting all the raw data in one study, or selecting all the studies with a single location

```
#### selecting a count rather than the data
dbGetQuery(myConnection, "SELECT count(*) from allrawdata where STUDY_ID=383")
```

```
##   count(*)
## 1       24
```

```
#### selecting a count rather than the data
dbGetQuery(myConnection, "SELECT count(STUDY_ID) from datasets where datasets.NUMBER_LAT_LONG=1")
```

```
##   count(STUDY_ID)
## 1             158
```

Because some fields are free text (such as Organism in *datasets*), entries may be slightly different (e.g. fish and reef fish, butterfly and Butterflies, coral and corals). To avoid issues with this, you can use the LIKE operator. This query will select all the distinct species and their study IDs for butterflies

```
result<-dbGetQuery(myConnection, "SELECT distinct GENUS_SPECIES, allrawdata.STUDY_ID FROM allrawdata inner join site on sit
e.STUDY_ID = allrawdata.STUDY_ID inner join datasets on datasets.STUDY_ID = allrawdata.STUDY_ID inner join species on specie
s.ID_SPECIES = allrawdata.ID_SPECIES where datasets.ORGANISMS like 'butter%'")

head(result)
```

```
##        GENUS_SPECIES STUDY_ID
## 1 Acherontia atropos       70
## 2  Agrius convolvuli       70
## 3    Agrotis ipsilon       70
## 4 Autographa bractea       70
## 5   Autographa gamma       70
## 6      Colias crocea       70
```

While this query selects the number of temperate fish species, and, unlike the query above, it does not include study ID because only the number of species is required

```
dbGetQuery(myConnection, "SELECT count(distinct GENUS_SPECIES) FROM allrawdata inner join site on site.STUDY_ID = allrawdat
a.STUDY_ID inner join datasets on datasets.STUDY_ID = allrawdata.STUDY_ID inner join species on species.ID_SPECIES = allrawd
ata.ID_SPECIES where datasets.TAXA = 'Fish' and site.CLIMATE='Temperate'")
```

```
##   count(distinct GENUS_SPECIES)
## 1                          2941
```

Disconnect

At the end of the session, it is important to disconnect from the database connection

```
dbDisconnect(myConnection)
```

```
## [1] TRUE
```

2.Using the csv file from the query

Here, we show how to interact with the full query to carry out some preliminary analyses using the raw data, and we also include code to perform sample-based rarefaction. In order to run the following examples, simply change the path to the downloaded csv file.

```
fullquery<-read.csv("/user/downloads/biotime/BioTIMEQuery_21_06_2017.csv") #### use the latest version from download site ##
##
```

Alternatively, you can load the query from the ZENODO repository

```
#####fullquery<-read.csv(url("http://some.where.net/data/BioTIMEQuery_21_06_2017.csv")) ##this will be code when we have the
```

The example below uses data from a single study *(Study ID 163)*.

```
query<-fullquery%>%
  filter(STUDY_ID == 163)
```

Sample-based Rarefaction code

```
# Maria Dornelas 09.06.2015
rarefysamples<-function(Year, SampleID, Species, Abundance, resamps) {
  #######################################################################
  # takes as input a  Year, SampleID, Species, Abundance and number of resamples
  # which should be in dataframe so that elements match
  # calculates turnover:
  # 1) between each year and the first year
  # 2) between pairs of adjacent years
  # 3) between each year and the lasy year of the time series
  # for the rarefied pooled samples
  ###########################################################################

  rareftab<-data.frame(array(NA,dim=c(0,3)))
  # getting vector with number of samples per year
  nsamples<-c()
  for(y in unique(Year)){
    nsamples<-c(nsamples, length(unique(SampleID[Year==y])))
  }
  t<-1
  minsample<-min(nsamples)
  for(repeats in 1:resamps){
    raref<-data.frame(array(NA,dim=c(1,3)))
    names(raref)<-c("Year","Species","Abundance")
    for(y in unique(Year)){
      #getting samples for this year
      samps<-unique(SampleID[Year==y])
      # re-sampling samples to equalize number of samples
      sam<-as.character(sample(samps,minsample,replace=T))
      # getting data that belongs to bootstraped samples
      rarefyear<-data.frame(SampleID[which(SampleID %in% sam & Year == y)],Species[which(SampleID %in% sam &Year
      ==y)],Abundance[which(SampleID %in% sam & Year == y)])
      names(rarefyear)<-c("SampleID", "Species", "Abundance")
      # calculating pooled abundances of each species to store
      spabun<-tapply(as.numeric(rarefyear[,3]),as.character(rarefyear[,2]),sum)
      spar<-data.frame(rep(y, length(spabun)),names(spabun),spabun, row.names=NULL)
      names(spar)<-c("Year","Species","Abundance")
      raref<-rbind(raref,spar)
    }
    # calculating year by species table of abundance
    rareftab<-rbind(rareftab,cbind(rep(repeats,dim(raref)[1]),raref))
  }
  return (rareftab)
}
```

Run the rarefaction code

```
TSrf<-list()
IDs<-unique(query$STUDY_ID)

for(i in 1:length(IDs)){
  data<-query[query$STUDY_ID==IDs[i],]
  TSrf[[i]]<-rarefysamples(data$YEAR, data$SAMPLE_DESC, data$GENUS_SPECIES, data$sum.allrawdata.ABUNDANCE, 1)
}
names(TSrf)<-IDs

rf<-do.call(rbind, TSrf)
rf<-data.frame(rf, ID=rep(names(TSrf), times=unlist(lapply(TSrf, nrow))))
rf<-rf[!is.na(rf$Year),-1]

#### prepare the rarefied output for diversity metric code
t1<-with(rf, tapply(Abundance, list(Year, Species, ID), sum))
t2<-unique(rf[, c("ID", "Year")])

#### produces a list of matrices for each study - in this case is only a single dataset
dsList<-list()

for(i in 1:dim(t1)[3]){
    id<-dimnames(t1)[[3]][i]
    a<-subset(t2, ID==id)$Year
    b<-t1[dimnames(t1)[[1]] %in% as.character(a),,i]
    dsList[[i]]<-data.frame(Year = rownames(b), b)
}

names(dsList) <- dimnames(t1)[[3]]

#### replacing NA with zero
for(i in 1:(length(dsList))){
    dsList[[i]][is.na(dsList[[i]])]<-0
}
```

Investigate biodiversity trends

```
#### Functions to calculate alpha and beta diversity metrics

####calculates species richness and total abundance
getAlpha<-function(x){
  data.frame(S=apply(x>0, 1, sum),
             N=apply(x, 1, sum))
  }

####calculates Jaccard and Morisita-Horn similarity indices
getBeta<-function(x, origin=1, consec=F){
  size<-dim(x)[1]-1
  ifelse(consec==T, id<-cbind(1:size, 1:size+1), id<-cbind(1:dim(x)[1], origin))
    output<-data.frame(Jaccard=as.matrix(1-vegdist(x, method="jaccard"))[id],
                       MorHorn=as.matrix(1-vegdist(x, method="horn"))[id])
  ifelse(consec==T, rownames(output)<-rownames(x)[-1], rownames(output)<-rownames(x))
    output
    }
```

```
#### get the alpha diversity metrics
alphaX<-lapply(dsList, function(x)getAlpha(x[,-1]))
alpha<-do.call(rbind,alphaX)
alpha$Year<-substring(rownames(alpha), nchar(rownames(alpha))-3)

#### get the beta diversity metrics
betaX<-lapply(dsList, function(x)getBeta(x[,-1]))
beta<-do.call(rbind, lapply(betaX, function(x)x[-1,]))
beta$Year<-substring(rownames(beta), nchar(rownames(beta))-3)
```

Function for the theme with no background grids but including axes titles

```
themeNoGridAxes <- function() {
   theme_bw()+
        theme(axis.text=element_text(size=16,color="black"),
        axis.title=element_text(size=16,face="bold"),
        legend.position="none",
        plot.title=element_text(size=18,face="bold", hjust=0.5),
        plot.background=element_blank(),
        panel.grid.major=element_blank(),
        panel.grid.minor=element_blank(),
        panel.border=element_blank(),
        axis.line=element_line(color="black")
    )
}
```

Plot these trends using ggplot

N.B. this code is tailored to produce a ‘nice’ plot with year breaks for STUDY_ID 163, it may need adjusted when running for other studies

```
#### plot species richness temporal trend with simple regression line
fitSR<-lm(alpha$S~alpha$Year)

srPlot<-ggplot(alpha, aes(x=Year, y=S))+geom_line(size=1, colour="gray30")+
  geom_point(aes(x=Year, y=S), colour="darkgreen", size=5)+
  scale_x_continuous(breaks=c(1993, 1995, 1997, 1999, 2001, 2003, 2005))+
  geom_abline(intercept=fitSR$coef[1], slope=fitSR$coef[2], colour="gray10", size=1, linetype=3)+
  themeNoGridAxes()+ylab("S")+xlab("Year")
srPlot
```

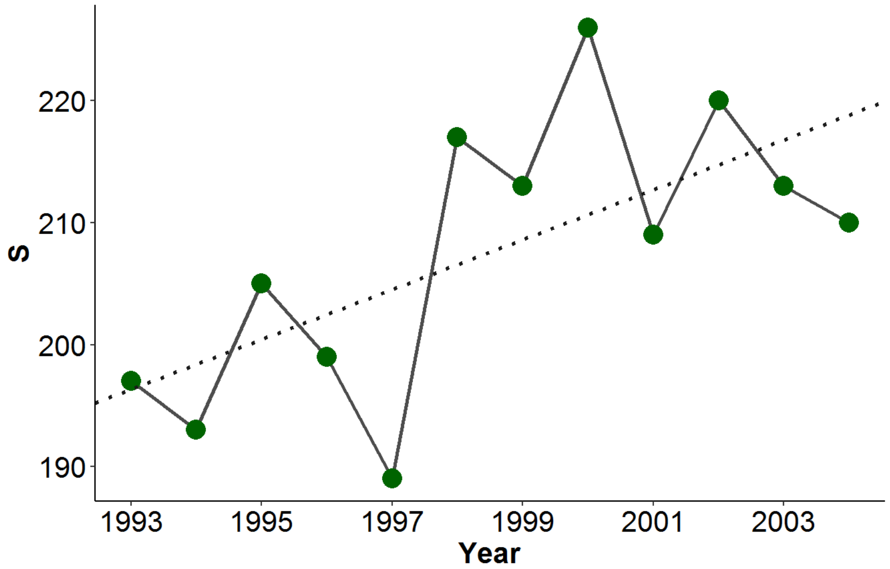


```
#### plot the Jaccard similarity temporal trend with a simple regression line
fitJ<-lm(beta$Jaccard~beta$Year)

jPlot<-ggplot(beta, aes(x=Year, y=Jaccard))+geom_line(size=1, colour="gray30")+
  geom_point(aes(x=Year, y=Jaccard), colour="dodgerblue", size=5)+
  scale_x_continuous(breaks=c(1993, 1995, 1997, 1999, 2001, 2003, 2005))+
  geom_abline(intercept=fitJ$coef[1], slope=fitJ$coef[2], colour="gray10", size=1, linetype=3)+
  themeNoGridAxes()+ylab("Jaccard Similarity")+xlab("Year")+ylim(0, 1)
jPlot
```

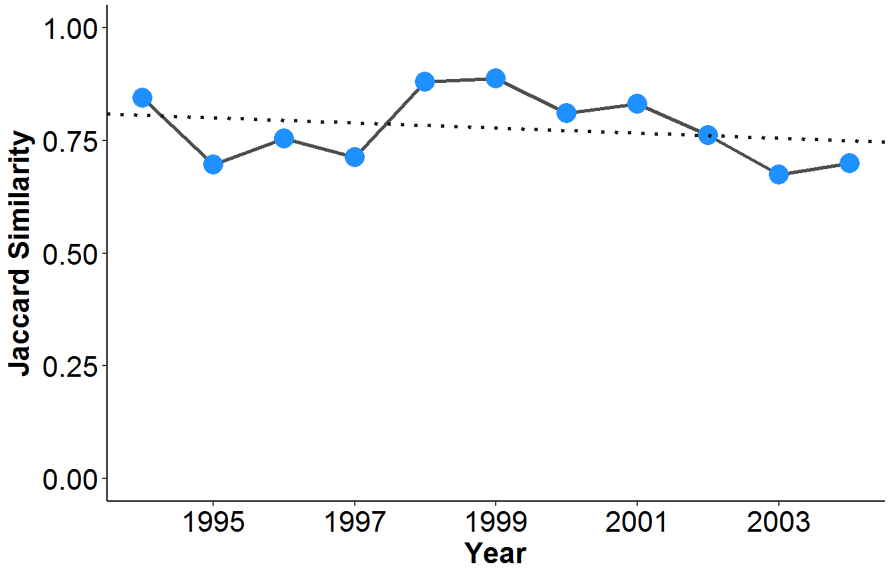

Producing maps and plots using the csv query

It is possible to create maps and plots, as shown in the first section of this document, using the metadata provided as a csv file.

Read the metadata csv file biotimeMeta (modify the path accordingly)

```
biotimeMeta<-read.csv("~/bioTIMEMetaData_20_06_2017.csv")
```

Use the same functions as above to draw a world map and overlay study locations

```
points<-drawWorld("y")+geom_point(data=biotimeMeta, aes(x=CENT_LONG, y=CENT_LAT, colour=TAXA, size=TOTAL), alpha=I(0.7))
points<-points+scale_colour_manual(values=taxaCol)+scale_size(range=c(3, 10))
points
```

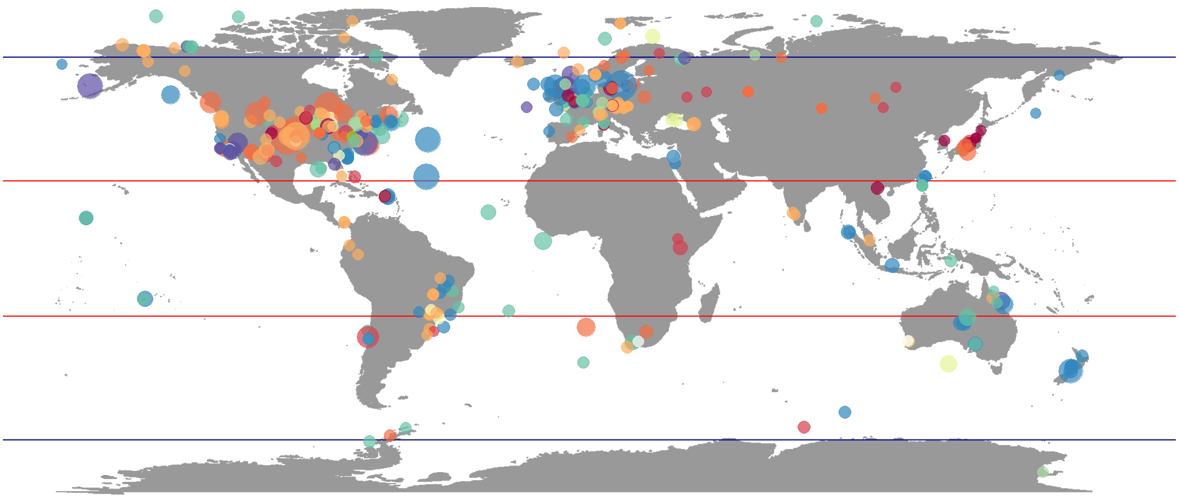

Note: You can download the [.Rmd file] “to insert link later” used to produce this document, but you will have to set up a connection with the SQL database, as well as have downloaded the csv files for both the query and the metadata, making sure that you modify all the paths accordingly.

erc
European Research Council
Established by the European Commission
