# Supplementary material for: BioTIME: A database of biodiversity time series for the Anthropocene
Source: Glob Ecol Biogeogr. 2018 Jul 24;27(7):760–86. doi: 10.1111/geb.12729 (PMC6099392; doi:10.1111/geb.12729)
Supplement: Supplementary file 2 — Supporting Information [file GEB-27-760-s002.pdf]

BioTIME: a database of biodiversity time series for the Anthropocene

Supplementary Information

Supplementary Figure 1-

Diagram illustrating database relationships
List of database tables with field descriptions and explanations.
Table of classifications of abundance and biomass held within BioTIME.

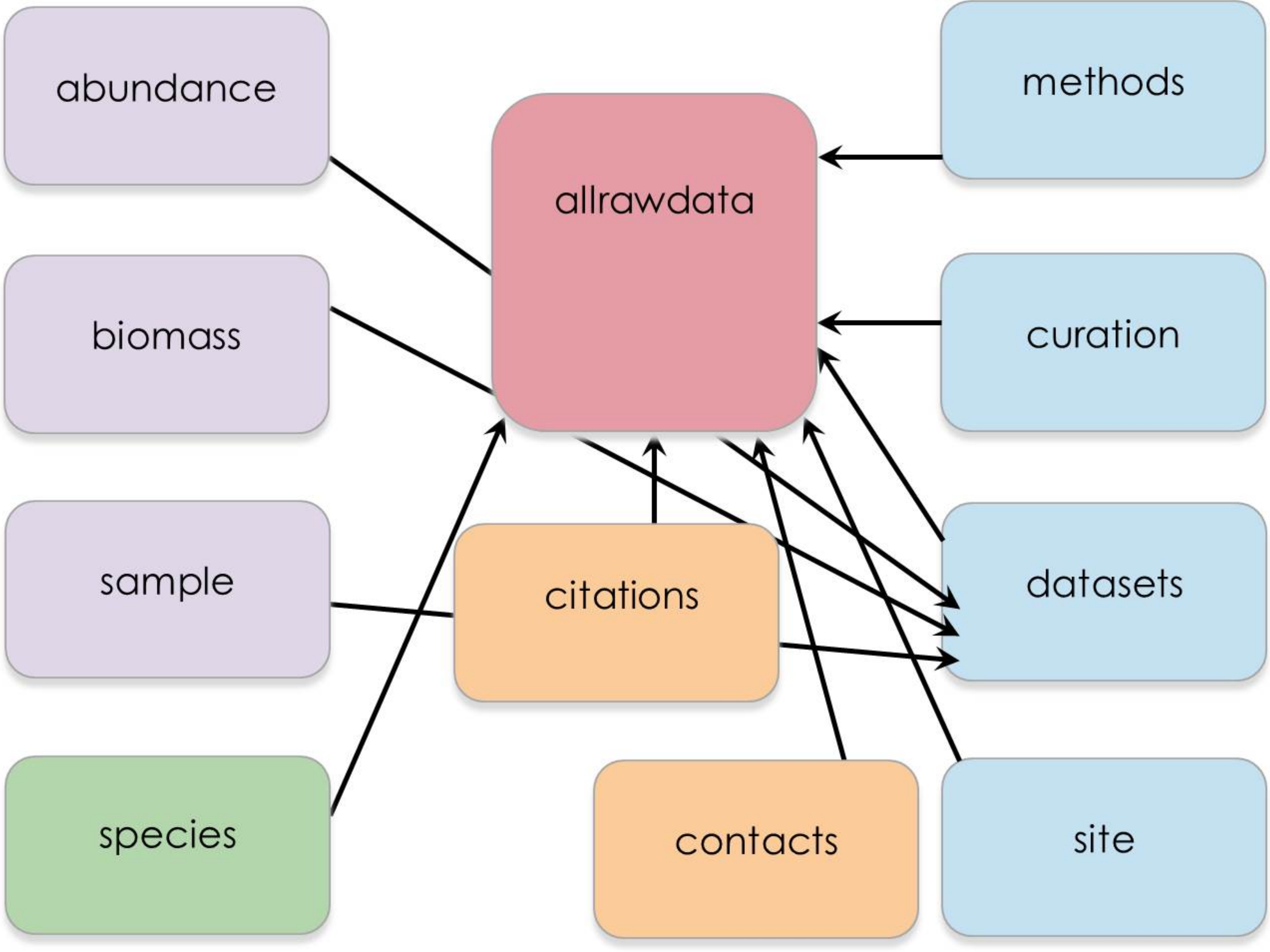

abundance
biomass
sample
species
allrawdata
citations
contacts
methods
curation
datasets
site

abundance

Column	Type	Null	Comments
ID_ABUNDANCE *(Primary)*	int(11)	No	Primary key for associated table, cannot be NULL
ABUNDANCE_TYPE	varchar(100)	Yes	Description of abundance measurement - classifications shown in table below

allrawdata

Column	Type	Null	Comments
ID_ALL_RAW_DATA *(Primary)*	int(11)	No	Primary key, cannot be NULL
ABUNDANCE	float	No	Numerical abundance of species in sample (record), may need to pool abundance of different life stages, sizes or sex.
BIOMASS	float	No	Biomass of species in sample (record), may need to pool abundance of different life stages, sizes or sex.
ID_SPECIES	int(11)	Yes	Numerical value linking to species table - where no previous entry for a species exists then it is added to the table and number assigned
SAMPLE_DESC	varchar(200)	No	The sample ID provided from the original data (if any), or a concatenation of relevant fields to determine the spatial and temporal sampling event; e.g. latitude_longitude_year_month_day_depth
PLOT	varchar(150)	Yes	Plot identifier (if any), this can be name of quadrat/plot/site or a concatenation of plot with separate areas, e.g. PLOT1/Q7, PLOT1/Q8
LATITUDE	decimal(10,6)	No	Actual latitude of record's location, e.g. -7.89
LONGITUDE	decimal(10,6)	No	Actual longitude of record's location, e.g. 16.988
DEPTH	float	Yes	Depth or elevation in metres (if available)
DAY	int(11)	Yes	Day of month in figures, e.g. 1 - 31
MONTH	int(11)	Yes	Month of year in figures, e.g. 1 - 12
YEAR	int(11)	Yes	Year in figures, e.g. 1976
STUDY_ID	int(11)	Yes	Study ID used to link to other metadata and rawdata

biomass

Column	Type	Null	Comments
ID_BIOMASS *(Primary)*	int(11)	No	Primary key for associated table, cannot be NULL
BIOMASS_TYPE	varchar(100)	Yes	Description of biomass measurement - classifications shown in table below

contacts

Column	Type	Null	Comments
ID_CONTACTS *(Primary)*	int(11)	No	Primary key, cannot be NULL
STUDY_ID	int(11)	Yes	Study ID used to link to other metadata and rawdata
CONTACT_1	varchar(500)	Yes	First point of contact
CONTACT_2	varchar(500)	Yes	Second (if any) point of contact
CONT_1_MAIL	varchar(60)	No	First contact email address
CONT_2_MAIL	varchar(60)	Yes	Second contact email address
LICENSE	varchar(200)	No	License type requested by data providers
WEB_LINK	varchar(200)	Yes	URL of any web links
DATA_SOURCE	varchar(250)	No	Original data source

curation

Column	Type	Null	Comments
ID_CURATION *(Primary)*	int(11)	No	Primary key, cannot be NULL
STUDY_ID	int(11)	Yes	Study ID used to link to other metadata and rawdata
LINK_ID	int(11)	Yes	ID of any associated datasets
COMMENTS	text	Yes	Comments describing any changes introduced during curation
DATE_STUDY_ADDED	varchar(50)	No	Date in text format - MMM-YY (Jan-17)

datasets

Column	Type	Null	Comments
ID_DATASETS *(Primary)*	int(11)	No	Primary key, cannot be NULL
STUDY_ID	int(11)	Yes	Study ID used to link to other metadata and rawdata
TAXA	varchar(50)	Yes	Taxa of study
ORGANISMS	varchar(200)	Yes	Organism of study
TITLE	varchar(450)	Yes	Title of study
AB_BIO	varchar(2)	Yes	Does study contain abundance, biomass or both (A, B or AB)
HAS_PLOT	varchar(10)	No	Does the study have a fixed plot - Y/N/S where Y=Yes, N==No and S=for some records (Some)
DATA_POINTS	smallint(6)	Yes	Number of years covered by study
START_YEAR	smallint(6)	Yes	First year of study
END_YEAR	smallint(6)	Yes	Final year of study
CENT_LAT	decimal(10,6)	No	Central latitudinal point of study - calculated using convex hull (centre point) e.g. 40.5785
CENT_LONG	decimal(10,6)	No	Central longitudinal point of study - calculated using convex hull (centre point) e.g. -9.87
NUMBER_OF_SPECIES	int(11)	Yes	Number of species in study
NUMBER_OF_SAMPLES	int(11)	Yes	Number of unique samples in study
NUMBER_LAT_LONG	int(11)	Yes	Number of geographic points (lat/longs) in study
TOTAL	int(11)	No	Total number of records within study
GRAIN_SIZE_TEXT	varchar(250)	Yes	Size of spatial grain in text if available
GRAIN_SQ_KM	double	No	Size of spatial grain in square km if available
AREA_SQ_KM	double	No	Area of study in square km
AB_TYPE	varchar(100)	No	Foreign key denoting a relationship with the abundance table
BIO_TYPE	varchar(100)	No	Foreign key denoting a relationship with the biomass table
SAMPLE_TYPE	varchar(250)	No	Foreign key denoting a relationship with the sample table

methods

Column	Type	Null	Comments
ID_METHODS *(Primary)*	int(11)	No	Primary key for associated table, cannot be NULL
STUDY_ID	int(11)	Yes	Study ID used to link to other metadata and rawdata
METHODS	text	Yes	Detailed method analysis including any treatments known
SUMMARY_METHODS	varchar(500)	Yes	Short description of method, e.g. plots, transects

sample

Column	Type	Null	Comments
ID_SAMPLE *(Primary)*	int(11)	No	ID found in allrawdata table to identify how the sample description has been concatenated
ID_TREAT	int(11)	Yes	Foreign key for treatment table (not in use as yet)
SAMPLE_DESC_NAME	varchar(200)	Yes	Descriptor of how ID is created, e.g. latitude_longitude_ year_plot_depth

site

Column	Type	Null	Comments
ID_SITE *(Primary)*	int(11)	No	Primary key, cannot be NULL
STUDY_ID	int(11)	Yes	Study ID used to link to other metadata and rawdata
REALM	varchar(11)	Yes	Realm of site - Marine, Terrestrial, Freshwater
CLIMATE	varchar(20)	Yes	Climate - Tropical, Temperate, Polar, Polar/temperate, Temperate/tropical, Global (encompassing more than two zones)
GENERAL_TREAT	varchar(200)	Yes	Description of treament (if any)
TREATMENT	varchar(200)	Yes	Treatment levels including control (if any)
TREAT_COMMENTS	varchar(250)	Yes	Description of each treament level (if any)
TREAT_DATE	varchar(100)	Yes	Dates of treatment (if any)
CEN_LATITUDE	decimal(10,6)	Yes	Central latitudinal point of site - calculated using convex hull (centre point)
CEN_LONGITUDE	decimal(10,6)	Yes	Central longitudinal point of site - calculated using convex hull (centre point)
HABITAT	varchar(100)	Yes	Description of habitat (general), possiblities include small ponds, coastal, small woodland etc.
PROTECTED_AREA	varchar(50)	Yes	TRUE/FALSE
AREA	float	Yes	Extent of site area in km2 (for current studies this is equivalent to study area (found in datasets))
BIOME_MAP	varchar(250)	Yes	Biome as listed on WWF site, at http://www.worldwildlife.org/biomes

species

Column	Type	Null	Comments
ID_SPECIES *(Primary)*	int(11)	No	ID found in allrawdata table to identify species
GENUS	varchar(100)	Yes	Genus (or if record has higher taxonomic resolution this can be Family, Order, etc.) e.g. Acer
SPECIES	varchar(100)	Yes	Specific epithet, e.g. circinatum
GENUS_SPECIES	varchar(100)	Yes	Concatenation of genus and species

Breakdown of abundance and biomass types

Broad classification of abundance		Abundance type
Presence/Absence		AggregatedPresence
		Presence
		Occurrence
Density		IndCountDec
		CountPerSqM
		DensityPer10Ha
		CountPer250m2
		DensityPer40Ha
		DensitySqM
		CountPerMinute
		CountPerHour
MeanCount		MeanCount
Count		AggregatedCount
		IndCountInt
		SummedCount
		Count
**Broad classification of biomass**		**Biomass type**
Weight		AboveGroundBiomass
		AggregatedWeight
		DryBiomass
		KiloPer250m2
		Weight
Volume		Biovolume
Cover		Cover
		PercentCover
Size		SnoutVentLength
		CountXestimatedSize
Relative biomass		PercentComposition

Supplementary Figure 2 –

Full BioTIME map

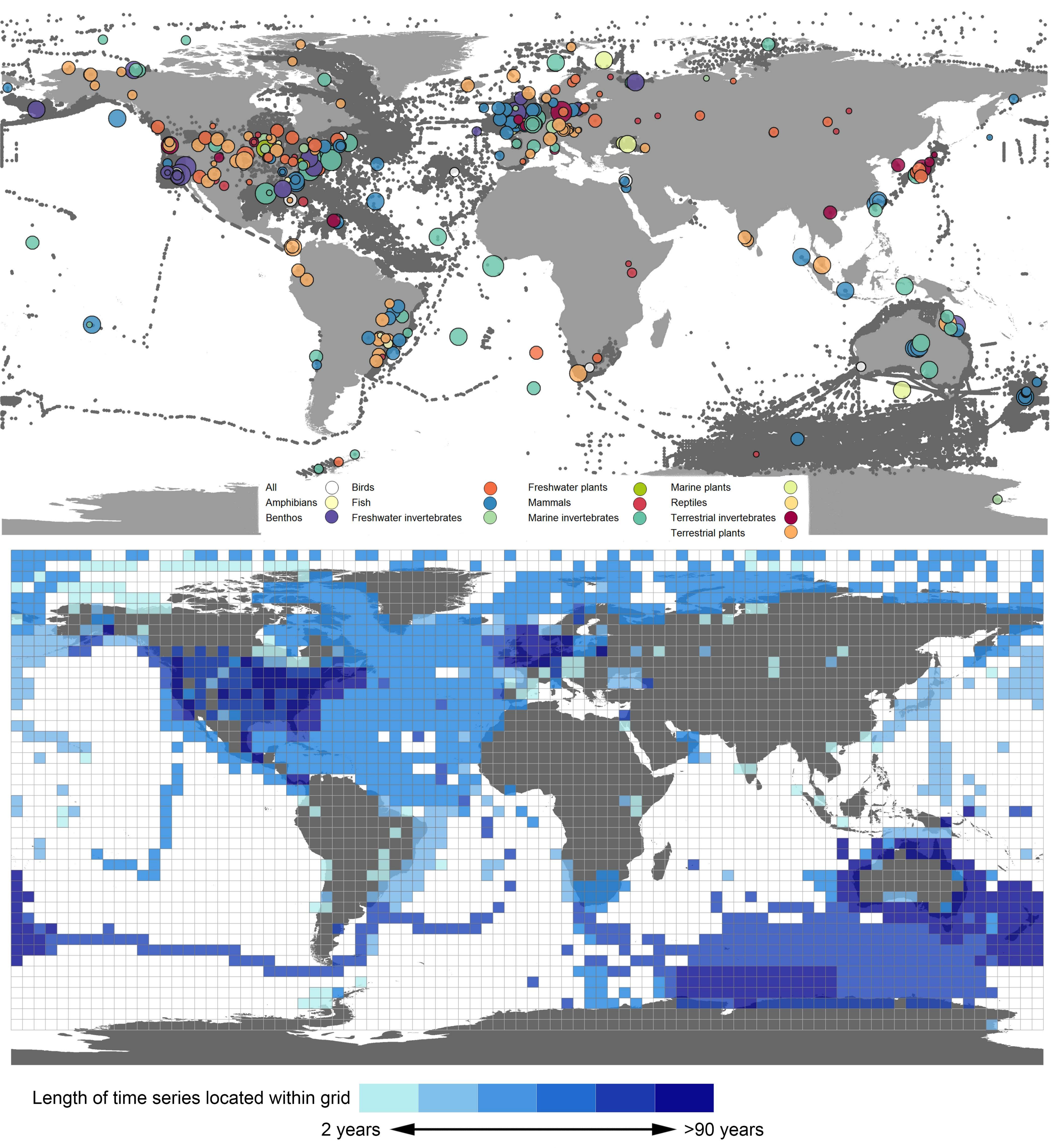
All
Amphibians
Benthos
Birds
Fish
Freshwater invertebrates
Freshwater plants
Mammals
Marine invertebrates
Marine plants
Reptiles
Terrestrial invertebrates
Terrestrial plants
Length of time series located within grid
2 years
>90 years
